# Maladaptive Modulations of NLRP3 Inflammasome and Cardioprotective Pathways Are Involved in Diet-Induced Exacerbation of Myocardial Ischemia/Reperfusion Injury in Mice

**DOI:** 10.1155/2016/3480637

**Published:** 2015-12-14

**Authors:** Raffaella Mastrocola, Massimo Collino, Claudia Penna, Debora Nigro, Fausto Chiazza, Veronica Fracasso, Francesca Tullio, Giuseppe Alloatti, Pasquale Pagliaro, Manuela Aragno

**Affiliations:** ^1^Department of Clinical and Biological Sciences, University of Turin, Regione Gonzole 10, Orbassano, 10043 Torino, Italy; ^2^Department of Drug Science and Technology, University of Turin, Corso Raffaello 33, 10125 Torino, Italy; ^3^Department of Life Sciences and Systems Biology, University of Torino, via Accademia Albertina 13, 10123 Torino, Italy; ^4^National Institute for Cardiovascular Research, via Irnerio 48, 40126 Bologna, Italy

## Abstract

Excessive fatty acids and sugars intake is known to affect the development of cardiovascular diseases, including myocardial infarction. However, the underlying mechanisms are ill defined. Here we investigated the balance between prosurvival and detrimental pathways within the heart of C57Bl/6 male mice fed a standard diet (SD) or a high-fat high-fructose diet (HFHF) for 12 weeks and exposed to cardiac *ex vivo* ischemia/reperfusion (IR) injury. Dietary manipulation evokes a maladaptive response in heart mice, as demonstrated by the shift of myosin heavy chain isoform content from *α* to *β*, the increased expression of the Nlrp3 inflammasome and markers of oxidative metabolism, and the downregulation of the hypoxia inducible factor- (HIF-)2*α* and members of the Reperfusion Injury Salvage Kinases (RISK) pathway. When exposed to IR, HFHF mice hearts showed greater infarct size and lactic dehydrogenase release in comparison with SD mice. These effects were associated with an exacerbated overexpression of Nlrp3 inflammasome, resulting in marked caspase-1 activation and a compromised activation of the cardioprotective RISK/HIF-2*α* pathways. The common mechanisms of damage here reported lead to a better understanding of the cross-talk among prosurvival and detrimental pathways leading to the development of cardiovascular disorders associated with metabolic diseases.

## 1. Introduction

Cardiovascular disorders associated with metabolic diseases are referred to as cardiometabolic diseases (CMDs). Despite the recent publication of several documents and papers suggesting clinical and social interventions to prevent CMDs and benefit subjects afflicted with these comorbidities, the identification of common mechanisms of disease is far from clear. A growing body of evidences indicates that excessive fatty acids and sugars intake affects the development and progression of cardiovascular diseases, including myocardial infarction, by increasing the local inflammatory response and, at the same time, by reducing the efficiency of protective responses that are usually activated by transient oxygen deprivation [1–3]. However, the underlying mechanisms leading to these impairments are complex, and a more thorough understanding is needed.

When exposed to an ischemic insult the cardiomyocytes easily switch from fatty acid (FA) oxidation towards glycolytic metabolism and increase glucose uptake to sustain ATP generation and support cardiac function. The loss of this metabolic flexibility is the main feature of a maladapted heart. For instance, mice with diet-induced obesity and exposed to daily repetitive brief-duration cardiac ischemia exhibited an early and profound downregulation of myocardial genes involved in FA oxidation, such as muscle-type carnitine palmitoyltransferase 1 (CPT-1m) and medium-chain acyl-coenzyme A dehydrogenase with respect to lean mice [2]. Besides, an excessive FA oxidation has been demonstrated to contribute to cardiac dysfunction in obesity and diabetes [4].

One of the most recently identified proinflammatory signaling pathways involved in CMDs is the NOD-like receptor pyrin domain containing 3 (Nlrp3) inflammasome, a large multimeric protein complex mediating the cleavage of inactive prointerleukin- (IL-) 1*β* and IL-18 into their active form [5]. We and others have recently demonstrated that activation of Nlrp3 inflammasome contributes to the development of heart failure and diet-induced renal dysfunction [6, 7], mainly by inducing IL-1*β* and IL-18 overproduction. These cytokines of the IL-1 family modulate the insulin-producing pancreatic *β*-cell function and act as inflammatory mediators in myocardial ischemia/reperfusion (IR) injury [8, 9]. Reactive oxygen species (ROS), which are produced during IR, may activate Nlrp3 inflammasome and all known Nlrp3 inflammasome activators generate ROS whereas ROS inhibitors block Nlrp3 inflammasome activation [10–12]. The ischemic injury may also evoke the transient activation of prosurvival signaling pathways and several studies demonstrate that the adaptations to hypoxic conditions are regulated by the relative activities of molecules such as Akt, extracellular-signal-regulated kinases (ERK), and glycogen synthase kinase- (GSK-) 3*β* that taken together constitute the so-called Reperfusion Injury Salvage Kinases (RISK) pathway [13, 14]. The activation of the RISK pathway confers cardioprotection against IR injury by avoiding the opening of the mitochondrial permeability transition pore at the onset of reperfusion [15]. Interestingly, this prosurvival RISK pathway signaling is less effective in animal models of obesity and insulin resistance [16]. For instance, hearts from mice fed a high-fat diet for 32 weeks showed compromised basal expression and activation of the prosurvival RISK pathway signaling compared to mice under normal diet [3].

Other protective pathways include the family of proteins that coordinates at the transcriptional level the cellular response to oxygen availability, mainly the hypoxia inducible factor- (HIF-) *α* [17]. HIF-1 and HIF-2 proteins are both increased in the peri-infarct area after myocardial infarction in rats and humans, and their powerful protection seems to implicate mechanisms modulating glucose uptake and utilization and preserving mitochondrial function [18–21]. HIF-2*α* expression occurs in remote areas from the infarct [18] and it is necessary to maintain normal lipid homeostasis, as constitutive HIF-2 activation in hepatocytes results in impaired fatty acid beta-oxidation, decreased lipogenic gene expression, and increased lipid storage capacity [22]. These data suggest a broader role for HIF-2*α* in the pathophysiology of several CMDs, including ischemic heart diseases.

Nevertheless, none of the above mentioned studies investigated the direct impact of dysmetabolic conditions (i.e., diet-induced insulin resistance) on the potential cross-talk among these different prosurvival and detrimental signaling pathways involved in ischemic myocardial dysfunction. Thus, we investigated the effects of an obesogenic/diabetogenic high-fat high-fructose (HFHF) diet on cardiac tolerance to IR challenging in mice and we validated the relevance of impaired pivotal intracellular mechanisms, in the heart, a key target organ of CMDs.

## 2. Materials and Methods

### 2.1. Animals and Dietary Manipulation

Male C57Bl/6j mice (Charles River Laboratories, Calco, LC, Italy) aged 4 weeks were randomly allocated into the following dietary regimens: a standard low-sugars low-fat diet (Control, *n* = 12) and a high-fat high-fructose diet (HFHF, *n* = 12), for twelve weeks. Standard diet (D12450K, Research Diet Inc., New Brunswick, NJ, USA) composition was as follows: 70% of calories in carbohydrates (55% from corn starch and 15% from maltodextrin), 10% of calories in fat (5% from soybean, 5% from butter). High-fat high-fructose diet (D03012907, Research Diet Inc.) composition was as follows: 35% of calories in carbohydrates (10% from maltodextrin and 25% from fructose), 45% of calories in fat (5% from soybean, 40% from lard). All groups received drink and food* ad libitum*.

The animal protocols followed in this study were approved by the local “animal use and care committee” and were in accordance with the European Directive 2010/63/EU on the protection of animals used for scientific purposes. All groups received drink and food* ad libitum*.

### 2.2. General Parameters

Body weight and food intake were recorded weekly. Fasting glycemia was measured at the start of the protocol and every 4 weeks by saphenous vein puncture using a glucometer (GlucoGmeter, Menarini Diagnostics, Firenze, Italy).

Systolic blood pressure and pulse rate were assessed at 11 weeks of dietary manipulation as the mean value of 10 consecutive measurements obtained in the morning using a tail-cuff sphygmomanometer (IITC; Life Sciences, Woodland Hills, CA).

### 2.3. *Ex Vivo* Ischemia/Reperfusion (IR) Injury

After 12 weeks of dietary manipulation, Control and HFHF mice were pretreated with 500 U heparin and anesthetized with sodium pentothal (50 mg/kg) by intraperitoneal injections before being culled by cervical dislocation. Hearts were rapidly excised, blood was rapidly collected from the thorax cavity, and plasma was isolated. The excised heart was rapidly perfused at 80 mmHg by the Langendorff technique with Krebs-Henseleit bicarbonate buffer containing (mM) NaCl 118, NaHCO_3_ 25, KCl 4.7, KH_2_PO_4_ 1.2, MgSO_4_ 1.2, CaCl_2_ 1.25, and glucose 11. The buffer was gassed with 95% O_2_ : 5% CO_2_. The temperature of the perfusion system was maintained at 37°C.

After a 30 min stabilization period, hearts were subjected to a protocol of IR, which consisted in 30 min of global no-flow, normothermic ischemia followed by a period of 60 min of reperfusion for hearts of both groups (IR Control and IR HFHF). Hearts of Control and HFHF mice, after stabilization, underwent 90 min perfusion only (Sham Control and Sham HFHF) and served as reference groups in western blot analysis (see the following).

The perfusate flowing out of the heart was collected and measured. Collected coronary effluent was used for measurement of lactate dehydrogenase (LDH) release. To assess the conditions of experimental preparation the coronary flow rate was determined by the amount of perfusate measured in a specific time period.

At the end of perfusion period, the heart was rapidly removed from the perfusion apparatus and divided into two parts by a coronal section (perpendicular to the long axis); while the apical part (less than 1/3 of ventricular mass) was frozen rapidly in liquid nitrogen and stored at −80°C and subsequently used for western blot and histological analysis, the basal part of ventricle was used for infarct size assessment.

### 2.4. Infarct Size Assessment

Infarct areas were assessed at the end of the experiments with the nitroblue tetrazolium (NBT) technique [23]. The basal part of the ventricles was dissected by transverse sections into two-three slices. Following 20 min of incubation at 37°C in 0.1% solution NBT (Sigma-Aldrich, St. Louis, MO, USA) in phosphate buffer, unstained necrotic tissue was carefully separated from stained viable tissue by an independent observer, who was unaware of the protocols. Since the ischemia was global and since we analyzed only the basal part of the ventricles, the necrotic mass was expressed as a percentage of the analyzed ischemic tissue (% of infarct size on ischemic tissue, %IS/IT).

### 2.5. Detection of Lactate Dehydrogenase (LDH) Release

The perfusion effluent was collected for 5 min immediately before ischemia and for the entire reperfusion period. LDH released from the heart was determined by spectrophotometric analysis at 340 nm [23].

### 2.6. Biochemical Parameters

Plasma lipid profile was determined by standard enzymatic procedures using reagent kits (triglycerides (TG), cholesterol, and high-density lipoproteins (HDL); Hospitex Diagnostics, Florence, Italy). Low-density lipoproteins (LDL) were calculated by the formula: total cholesterol − [HDL + (TG/5)]. Plasma insulin level was measured using an enzyme-linked immunosorbent assay (ELISA) kit (Mercodia AB, Uppsala, Sweden).

### 2.7. Oil Red Staining

Cardiac intramyocellular lipid accumulation was evaluated by Oil Red staining on 10 *μ*m apex cryostatic sections. Stained tissues were viewed under an Olympus Bx4I microscope (40x magnification) with an AxioCamMR5 photographic attachment (Zeiss, Gottingen, Germany).

### 2.8. Immunohistochemistry

GLUT-4 expression was assessed on 10 *μ*m apex cryostatic sections by immunohistochemistry. Endogenous peroxidases were inactivated by incubating sections for 5 min with 0.3% H_2_O_2_. Sections were then blocked for 1 h with 3% BSA in PBS. Thus, sections were incubated overnight with rabbit anti-GLUT-4 primary antibody (Abcam, Cambridge, UK) followed by HRP-conjugated secondary antibodies. Sections were digitised with a high resolution camera (Zeiss) at 20x magnification.

### 2.9. Western Blot Analysis

Total proteins extracts were obtained from 10% (w/v) apex homogenates in RIPA buffer (0.5% Nonidet P-40, 0.5% sodium deoxycholate, 0.1% SDS, 10 mmol/L EDTA, and protease inhibitors). Protein content was determined using the Bradford assay. Protein extracts were stored at −80°C until use. Equal amounts of proteins were separated by SDS-PAGE and electrotransferred to nitrocellulose membrane. Membranes were probed with goat anti-*α*-myosin heavy chain (*α*-MHC), goat anti-*β*-MHC, rabbit anti-carnitine palmitoyltransferase (CPT) 1m, mouse anti-succinate dehydrogenase (SDH), anti-glucose transport- (GLUT-) 4 primary antibody (Abcam, Cambridge, UK), rabbit anti-phospho-insulin receptor 2 (IRS2^Ser270^), and mouse anti-IRS-2 (Cell Signaling, Danvers, MA, USA), rabbit anti-Nlrp3 (Epitomics, Burlingame, CA, USA), rabbit anti-caspase-1, rabbit anti-pERK1/2, rabbit anti-ERK, rabbit anti-pAkt^Ser473^, rabbit anti-Akt, rabbit anti-pGSK-3*β* ^Ser9^, rabbit anti-GSK-3*β*, mouse anti-hypoxia inducible factor- (HIF-) 2*α*, and goat anti-hydroxynonenal (HNE) (Novus Biologicals, Abingdon, UK) primary antibodies, followed by incubation with appropriated HRP-conjugated secondary antibodies (Bio-Rad Laboratories, Hercules, CA, USA). Proteins were detected with ECL detection system (ECL Clarity, Bio-Rad Laboratories, Hercules, CA, USA) and quantified by densitometry using analytic software (Quantity-One, Bio-Rad Laboratories, Hercules, CA, USA). Results were normalized with respect to *α*-tubulin densitometric value.

### 2.10. Materials

Unless otherwise stated, all compounds were purchased from the Sigma-Aldrich Company Ltd. (St. Louis, Missouri, USA). Antibodies were from Santa Cruz Biotechnology (Santa Cruz, CA, USA).

### 2.11. Statistical Analysis

All values are expressed as means ± SD. The Shapiro-Wilk test was used to assess the normality of the variable distributions. One-way ANOVA followed by Bonferroni's post hoc test was adopted for comparisons among selected pairs of groups: Control Sham versus Control IR; Control Sham versus HFHF Sham; Control IR versus HFHF IR; HFHF Sham versus HFHF IR. A *P* value < 0.05 was considered statistically significant. Statistical tests were performed with GraphPad Prism 6.0 software package (GraphPad Software, San Diego, CA, USA).

## 3. Results

### 3.1. General Parameters

After 12 weeks of HFHF diet, mice showed a marked increase in total body weight, accompanied by reduced heart-to-body weight ratio and a significant increase in plasma fasting levels of glucose, insulin, triglycerides, and cholesterol, when compared to Control mice (Table 1). In contrast, HFHF diet did not affect systolic blood pressure or pulse rate (data not shown).

### 3.2. Diet-Induced Cardiac Adaptation

**Figure 1:**
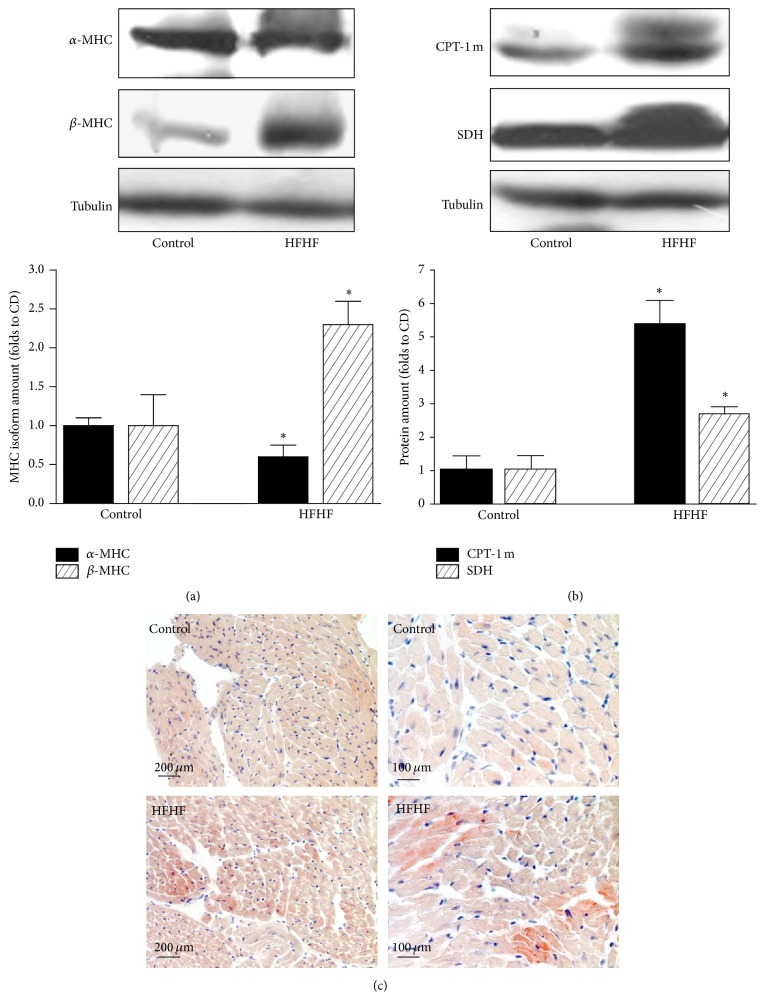
Cardiac metabolic adaptations to diet. Representative western blotting showing cardiac levels of *α*-MHC and *β*-MHC isoforms (a) and of markers of oxidative metabolism CPT-1 and SDH (b) assessed after 12 weeks of Control or HFHF diet in heart apex extracts. Histograms report densitometric analysis of 10–12 mice per group normalized for the corresponding tubulin content. ^*∗*^ *P* < 0.05 versus Control. (c) Representative 20x/40x magnification photomicrographs of heart apex sections from Control or HFHF diet mice showing cardiac intramyocellular lipid accumulation by Oil Red O staining.

As a shift in myosin heavy chain (MHC) isoform content from *α* to *β* is known to contribute to the development of heart failure, we measured the cardiac expression of the two functionally distinct cardiac MHC isoforms by western blotting analysis. As shown in Figure 1(a), a marked increase in *β*-MHC expression paralleled by a slight reduction in expression of *α*-MHC was recorded in the hearts of mice chronically exposed to the HFHF diet in comparison to hearts from Control mice, thus confirming a significant MHC isoform shift. This effect was associated with dramatic increase of CPT-1m and SDH, two markers of oxidative metabolism, following HFHF diet exposure (Figure 1(b)).

In addition, Oil Red O staining on heart sections revealed an intramyocellular lipid accumulation in HFHF mice that was not detected in Control mice (Figure 1(c)).

### 3.3. Infarct Area and LDH Release Increased in HFHF Hearts

**Figure 2:**
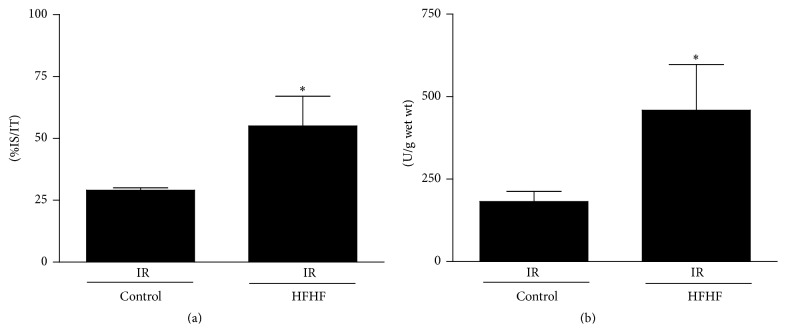
Infarct area and LDH release. Hearts from mice fed for 12 weeks with Control or HFHF diet, exposed to 30-minute ischemia plus 60-minute reperfusion. (a) Infarct size in the basal part of the ventricle after IR exposition is expressed as a percentage of ischemic tissue (%IS/IT). (b) LDH release in the perfusion effluent during the IR was expressed as units per mg of wet tissue weight. ^*∗*^ *P* < 0.05 versus Control.

When mice underwent myocardial IR, the IR infarct size recorded in the HFHF group was doubled with respect to that recorded in the Control IR (Figure 2(a)). Total LDH release during the 60 min of reperfusion corroborated this observation as it reached a 2.5-fold increase in HFHF IR group when compared to the Control IR value (Figure 2(b)).

### 3.4. Effects of HFHF and IR Injury on Cardiac GLUT-4 Translocation and Expression and IRS-2 Activation

**Figure 3:**
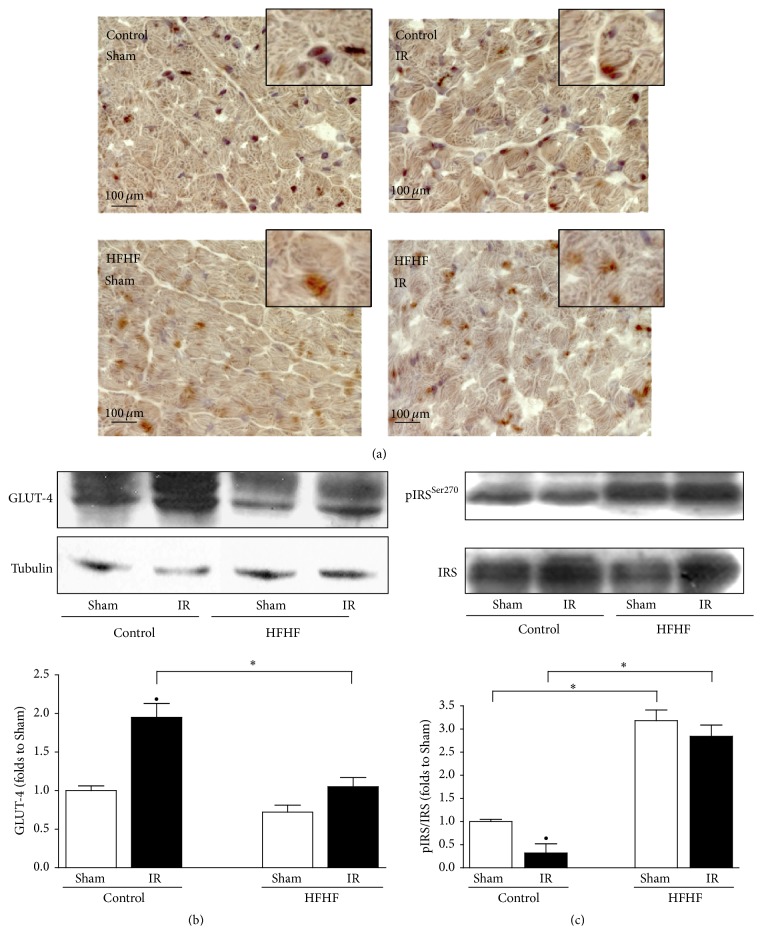
Localization and expression of GLUT-4 and IRS-2 activation in the mouse heart. Representative 40x magnification photomicrographs of heart apex sections and western blotting analysis on heart apex extracts from Control or HFHF diet mice, exposed or not to IR, showing GLUT-4 localization (a) and expression (b). Representative western blotting for cardiac levels of total IRS-2 and Ser270 phosphorylation performed on heart apex extracts from Control or HFHF diet mice, exposed or not to IR (c). Histogram reports densitometric analysis of the phosphorylated-to-total form ratio of 5-6 mice per group. ^*∗*^ *P* < 0.05 versus Control; ^•^ *P* < 0.05 versus Sham.

Immunohistochemistry and western blotting analysis showed that translocation from cytosol to membranes (Figure 3(a)) and expression (Figure 3(b)) of GLUT-4 were both reduced by HFHF diet, thus indicating a diet-induced insulin resistance of the cardiomyocytes. This was confirmed by the markedly increased phosphorylation rate of IRS-2 that inactivates insulin signaling in HFHF hearts assessed by western blotting (Figure 3(c)). The IR challenge induced the increase in GLUT-4 translocation and IRS-2 activation in Control mice, while in HFHF-fed mice IR did not significantly modify GLUT-4 expression and translocation or IRS-2 phosphorylation rate, with respect to HFHF Sham (Figures 3(a), 3(b), and 3(c)).

### 3.5. Effects of HFHF and IR Injury on Cardiac Lipid Peroxidation and Mitochondrial Oxidative Stress

**Figure 4:**
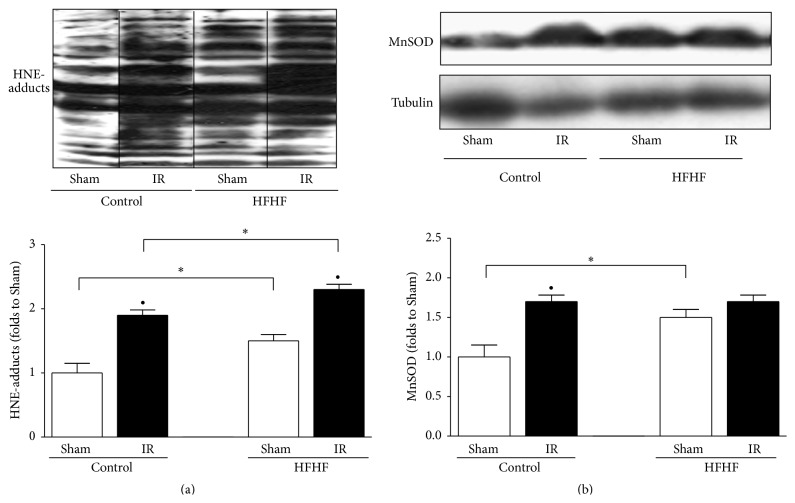
Oxidative stress parameters. Representative western blotting showing cardiac levels of HNE-protein adducts (a) and MnSOD (b) assessed on heart apex extracts from Control or HFHF diet mice, with or without IR. Histograms report densitometric analysis of 5-6 mice per group normalized, respectively, for the corresponding tubulin content. ^*∗*^ *P* < 0.05 versus Control; ^•^ *P* < 0.05 versus Sham.

As shown by western blotting analysis, HFHF diet induced a significant increase in HNE-protein adducts in both Sham and IR experimental conditions in comparison to Control mice, thus demonstrating a robust diet-induced production of lipid peroxidation products (Figure 4(a)). Interestingly, hearts exposure to the IR challenge evoked a further increase in the levels of lipid peroxidation products in both Control and HFHF groups (Figure 4(a)). When the expression of the antioxidant MnSOD enzyme was measured, a significant upregulation was recorded following chronic treatment with HFHF diet. In contrast, IR injury induced MnSOD expression in the heart of Control mice but not in the heart of HFHF mice, thus suggesting that the antioxidant defense in HFHF hearts could not be further increased by IR (Figure 4(b)).

### 3.6. NLRP3 Inflammasome Complex Activation

**Figure 5:**
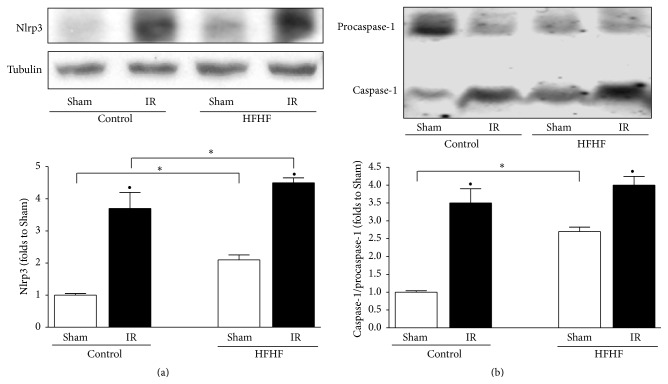
Inflammasome expression and activation in the mouse heart. Representative western blotting showing cardiac levels of Nlrp3 (a), the best characterized element of inflammasome complex, and of downstream activation of caspase-1 (b) assessed on heart apex extracts from Control or HFHF diet mice, with or without IR. Histograms report densitometric analysis of 5-6 mice per group normalized, respectively, for the corresponding tubulin content or the procaspase-1 content. ^*∗*^ *P* < 0.05 versus Control; ^•^ *P* < 0.05 versus Sham.

As assessed by western blot analysis, IR induced a strong upregulation of both Nlrp3 inflammasome and activated caspase-1 in the heart samples from Control and HFHF mouse, although basal expression levels of Nlrp3 inflammasome and activated caspase-1 in Sham HFHF mouse hearts were already drastically higher than those recorded in Sham Control hearts (Figure 5).

### 3.7. RISK Pathway Activation

**Figure 6:**
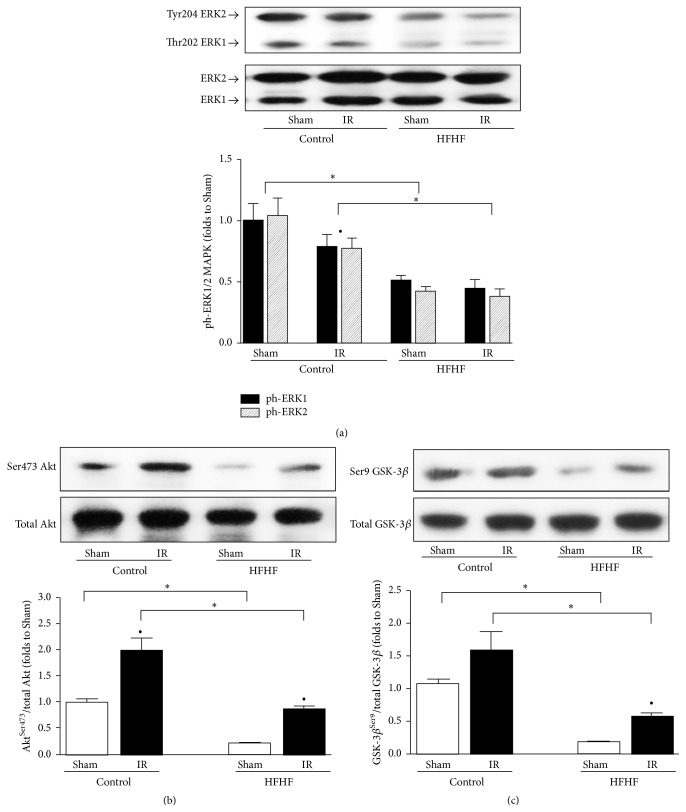
Prosurvival RISK pathway activation in the mouse heart. Representative western blotting for cardiac levels of total ERK1/2 expression and Thr202/Tyr204 phosphorylation, respectively, (a), total Akt protein expression and Ser473 phosphorylation (b), and total GSK-3 protein expression and Ser9 phosphorylation (c) performed on heart apex extracts from Control or HFHF diet mice, exposed or not to IR. Histograms report densitometric analysis of the phosphorylated-to-total form ratio of 5-6 mice per group. ^*∗*^ *P* < 0.05 versus Control; ^•^ *P* < 0.05 versus Sham.

In Control diet hearts, IR challenging did not induce significant variations of phospho-ERK1/2 (Figure 6(a)), while a marked increase in both phospho-Akt/Akt (Figure 6(b)) and phospho-GSK-3*β*/GSK-3*β* ratios (Figure 6(c)) was observed. The basal levels of ERK1/2, Akt, and GSK-3*β* phosphorylation in the hearts of the HFHF group were significantly lower than those reported in the Control diet hearts. The IR-induced upregulation of enzyme phosphorylation following dietary manipulation still remained lower than those evoked by the same insult in Control mice and no effects were recorded on ERK1/2 expression and phosphorylation.

### 3.8. HIF-2*α* Activation

**Figure 7:**
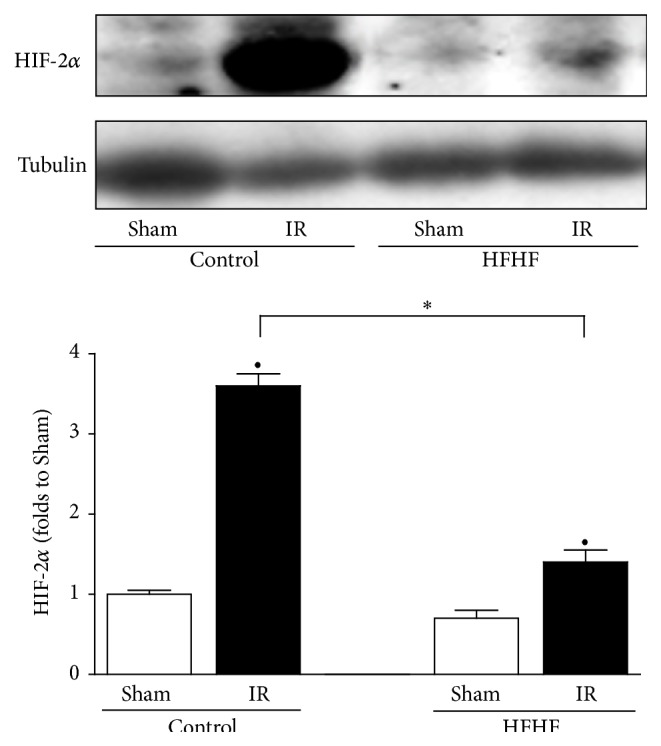
HIF-2*α* expression in the mouse heart. Representative western blotting for cardiac levels of HIF-2*α* performed on heart apex extracts from Control or HFHF diet mice, exposed or not to IR. Histograms report densitometric analysis of 5-6 mice per group normalized for the corresponding tubulin content. ^*∗*^ *P* < 0.05 versus Control; ^•^ *P* < 0.05 versus Sham.

Twelve weeks of HFHF diet led to a slight but not significant reduction in HIF-2*α* expression in heart extracts. Hearts from Control mice exposed to IR underwent a robust induction of HIF-2*α* expression. In contrast, in the hearts of HFHF mice, the expression level of HIF-2*α* was reduced by HFHF exposure and reported to Control Sham value by IR, remaining significantly lower than in IR Control hearts (Figure 7).

## 4. Discussion

In this study, we demonstrated that chronic feeding with an HFHF diet induces a maladaptive response in cardiac tissue, as shown by the *α*- to *β*-MHC isoform shift, the increased expression of markers of mitochondrial oxidative metabolism, such as CPT-1m and SDH, and the reduced cardiac glucose uptake. Interestingly, despite increased markers of oxidative metabolism, HFHF diet was associated with intramyocytes triglyceride accumulation, suggesting that the tightly regulated process of FA uptake and utilization was perturbed. These effects were paralleled by a robust increase in markers of mitochondrial oxidative stress and lipid peroxidation in the hearts of HFHF mice. Similar results have been previously documented in different experimental models of diabetic cardiomyopathy [24, 25] and the effect of myocardial lipid accumulation on the impairment of systolic cardiac performance is well known [26]. Although there are contrasting data on cardiac postischemic outcomes in models of diet-induced dysmetabolism [27], we here documented worsening of cardiac IR effects in animals exposed to the obesogenic/diabetogenic diet. To better elucidate the impact of dietary manipulation on myocardial IR injury, we assessed the expression and activation of IR-related signaling pathways. One of the most widely studied protective cascades which is involved in mediating the protective effects of many cardioprotective interventions is the RISK pathway [16, 28, 29]. Interestingly, the activities of members of the RISK pathway, including Akt, ERK1/2, and GSK-3*β*, are often impaired in conditions of diabetes, obesity, insulin resistance, and hypercholesterolemia [3, 16, 29, 30]. In this context, we previously reported that high-fat high sugar diets lead to reduced Akt-mediated insulin signaling [21, 31]. Here we show that the protective myocardial RISK pathway is upregulated by cardiac IR challenging and, most notably, this upregulation is lost in HFHF mice. These results suggest that the reduced RISK activation contributes to the exacerbated myocardial injury in HFHF mice. This is consistent with findings of other authors showing that the presence of metabolic derangements abrogates the protective preconditioning-induced activation of RISK pathway [16, 30, 32]. A consequence of the RISK pathway activation in the early response to cardiac oxygen deprivation is the increased expression of HIF-2*α* in remote areas from the infarct [18]. HIF-2*α* regulates key processes of long-term adaptation and maintains mitochondrial homeostasis by regulating production of antioxidant enzymes [33]. A recent research study reported the association between reduced Akt signaling and impaired HIF proteins activity [34]. Moreover, recent studies indicate that HIF-2*α* directly regulates IRS-2 transcription in diabetic mice both* in vivo* and in primary hepatocytes, thus improving insulin sensitivity and increasing Akt activation [35, 36]. Our results further extend this observation, demonstrating for the first time that an HFHF diet negatively impacts cardiac HIF-2*α* expression during IR, thus compromising the heart response to IR and the related activation of the insulin signalling.

Intriguingly, the diet-induced inhibition of protective signaling pathways was associated with a robust increase in myocardial protein levels of Nlrp3 inflammasome. The key role of the Nlrp3 inflammasome as central mediator in the inflammatory response to tissue injury during either myocardial infarction or insulin resistance is already known [7, 37, 38]. Strong correlations between the expression of Nlrp3 inflammasome-related genes and insulin resistance have been recently reported in obese male subjects with impaired glucose tolerance and in type 2 diabetic patients [39, 40]. Besides, genetic or pharmacological inhibition of Nlrp3 inflammasome reduces infarct size and limits the development of diet-induced obesity [41, 42]. However, the present study is, to the best of our knowledge, the first one demonstrating that the upregulation of Nlrp3 protein evoked by IR injury is drastically higher in the presence of a diet-induced metabolic derangement. These findings suggest a potential association between increased activity of Nlrp3 inflammasome following metabolic derangements and enhanced susceptibility to a myocardial ischemic insult. Overall, the diet- and IR-induced redox imbalance may represent the key event leading to the signaling pathways modifications. Indeed, a previous study showed that HIF-2*α* knockout mice show multiorgan damage due to ROS overproduction [43], and genetic deletion of HIF-2*α* resulted in increased levels of oxidative stress markers [33]. In keeping with these findings, we observed a reduced expression of HIF-2*α* and MnSOD in HFHF mice exposed to IR, which could account for the dramatic increase in HNE-adducts. Similarly, mitochondrial oxidative stress is one of the main stimuli triggering Nlrp3 activation [44–46]. For instance, HNE treatment of retinal pigment epithelial cells strongly induces Nlrp3 expression, leading to IL-1*β* and IL-18 production [47]. We may, thus, speculate that the oxidative unbalance due to impairments in RISK/HIF-2*α* pathways can worsen the proinflammatory response triggered by Nlrp3 activation. However, further investigations are required to better elucidate the intricate mechanisms of cross-talk among signaling pathways operational in the pathogenesis and potentially also the resolution of CMDs.

The experimental model here proposed allows us to study the intrinsic capacity of the myocardium to afford the IR challenge in a strictly controlled environment, avoiding extracardiac influences and the possible effect of temperature and collateral flow variations. However, we are aware of some limitations of the present study, including the lack of hemodynamic and functional data of postischemic myocardium and the impossibility to dissect between the redox effects on postischemic necrosis and stunning. Future* ad hoc* studies, with implemented technologies, are required to clarify these aspects.

In conclusion, our results clearly demonstrate that a high-fat high-fructose diet alters different signaling pathways involved in cardiac IR injury. While elements of cardioprotective pathways are downregulated, those of inflammatory processes are upregulated by HFHF diet and IR injury is exacerbated by these maladaptive pathway modulations. These results offer further improvements of our understanding of the link between cardiovascular and metabolic injuries. However, further studies are needed to better clarify the reciprocal interaction of these pathways within CMDs pathogenesis, thus allowing the identification of new therapeutic targets for improving postischemic recovery in obese/diabetic patients.

## Figures and Tables

**Table 1:** General parameters of mice after 12 weeks of Control or high-fat high-fructose (HFHF) diets.

	Control (*n* = 10)	HFHF (*n* = 12)
Body weight increase (g)	8.5 ± 1.6	14.7 ± 3.2^*∗∗∗*^
Heart weight (% of body w.)	0.45 ± 0.02	0.35 ± 0.06^*∗∗*^
Plasma glucose (mg/dL)	73 ± 19	139 ± 13^*∗∗∗*^
Plasma insulin (mg/mL)	85.8 ± 5.3	106.8 ± 6.8^*∗∗∗*^
Plasma TG (mg/dL)	32.8 ± 7.8	66.5 ± 25.5^*∗∗∗*^
Plasma Chol. (mg/dL)	77.2 ± 5.7	97.2 ± 8.2^*∗∗*^

Data are means ± SD. ^*∗∗*^ *P* < 0.01, ^*∗∗∗*^ *P* < 0.005 versus Control.
